# Dynamic Modeling and Experimental Validation of a Water Hydraulic Soft Manipulator Based on an Improved Newton—Euler Iterative Method

**DOI:** 10.3390/mi13010130

**Published:** 2022-01-14

**Authors:** Yinglong Chen, Qiang Sun, Qiang Guo, Yongjun Gong

**Affiliations:** 1 Naval Architecture and Ocean Engineering College, Dalian Maritime University, Dalian 116000, China; s2549724961@163.com (Q.S.); guoqiang01@163.com (Q.G.); yongjungong@163.com (Y.G.); 2 State Key Laboratory of Fluid Power and Mechatronic Systems, Zhejiang University, Hangzhou 310027, China

**Keywords:** Newton–Euler iterative method, dynamic modeling, water hydraulic system, soft manipulator

## Abstract

Compared with rigid robots, soft robots have better adaptability to the environment because of their pliability. However, due to the lower structural stiffness of the soft manipulator, the posture of the manipulator is usually decided by the weight and the external load under operating conditions. Therefore, it is necessary to conduct dynamics modeling and movement analysis of the soft manipulator. In this paper, a fabric reinforced soft manipulator driven by a water hydraulic system is firstly proposed, and the dynamics of both the soft manipulator and hydraulic system are considered. Specifically, a dynamic model of the soft manipulator is established based on an improved Newton–Euler iterative method, which comprehensively considers the influence of inertial force, elastic force, damping force, as well as combined bending and torsion moments. The dynamics of the water hydraulic system consider the effects of cylinder inertia, friction, and water response. Finally, the accuracy of the proposed dynamic model is verified by comparing the simulation results with the experimental data about the steady and dynamic characteristics of the soft manipulator under various conditions. The results show that the maximum sectional error is about 0.0245 m and that the maximum cumulative error is 0.042 m, which validate the effectiveness of the proposed model.

## 1. Introduction

In recent years, soft robots have gradually become a research hotspot in the field of robotics [1,2,3,4,5]. Different from the rigid links and joints of traditional rigid robots, the advantages of soft robots are mainly reflected in the flexibility of their materials and functions, which gives soft robots infinite freedom of body. Soft robots can produce passive deformation according to the environment to complete operations in complex and unstructured environments, greatly making up for the deficiency of traditional rigid robots [6,7,8,9].

Soft robots have good human–robot interaction performance due to their compliant characteristics [10]. However, due to the lack of a rigid structure, their stiffness is limited, and the attitude of soft robots is difficult to predict under the conditions of an external load. Therefore, the model research of soft robots is very necessary and important. At present, an effective kinematics model of soft robots has been developed, and a constant curvature model has been widely used [11,12]. Marchese and Rus of MIT proposed a simplified continuous piecewise constant curvature model for a soft manipulator [13]. Lukas et al. from King’s University London established a forward and inverse kinematics model of an FEA-driven soft manipulator via Timoshenko’s theory [14]. The Beijing University of Aeronautics and Astronautics Wen Li team analyzed the mapping relationship between the fluid driving pressure and the elongation of the driving unit, and established a kinematics model of the manipulator arm while ignoring the radial expansion of the driving unit [15]. Dalian Maritime University improved the constant curvature model and proposed a soft manipulator kinematics modeling method based on the curvature division of the contact point [16]. In addition, in order to further study and derive the kinematics of the soft manipulator, a plane static mechanics model and a space static mechanics model were also developed. These methods include beam mechanics [17,18], a Cosserat rod model [19,20], elliptic integral kinematics [21], modal approximation [22], a lumped model [23] and so on.

However, compared with the kinematics model, the study of the dynamic model still lacks progress. At present, the dynamic model in the field of the soft manipulator mainly adopts the Euler—Lagrange method [24,25,26], Kane method [27], and dynamics analysis method based on the Cosserat theory [28,29,30]. Mahl et al. proposed a bellows driven soft manipulator dynamic model based on the Euler—Lagrange method [31]. Wang et al. proposed an underwater dynamic model of a tethered soft robot based on the Kane method [32]. The precision of the model is high, but it also requires more complex calculations and takes more time.

Compared with dynamic modeling methods such as the Euler—Lagrange method and Cosserat theory, it is easier to use the Newton—Euler iterative method to establish the derivation equation of the dynamic model of the soft manipulator, and it is more convenient and efficient to design the model-based controller based on the Newton-Euler model. However, there are still few studies on the dynamic modeling of soft manipulators based on the Newton-Euler iterative method [33]. In order to expand the dynamics research on the soft manipulator, this paper establishes a dynamic model of the soft manipulator based on the Newton—Euler method. In this model, the influences of environmental resistance, inertial and elastic forces, damping forces, and combined moments of bending and torsion are considered comprehensively.

This paper is divided into four parts. In Section 1, the importance of the research is expounded upon and the research content is briefly introduced. In Section 2, dynamic models of the soft manipulator and hydraulic system are establish respectively. In Section 3, the dynamic model is simulated. In Section 4, an experimental platform is built to verify the theoretical model experimentally. Finally, the conclusion of the research is drawn in Section 5.

## 2. Dynamic Model

### 2.1. Kinematics

**Figure 1:**
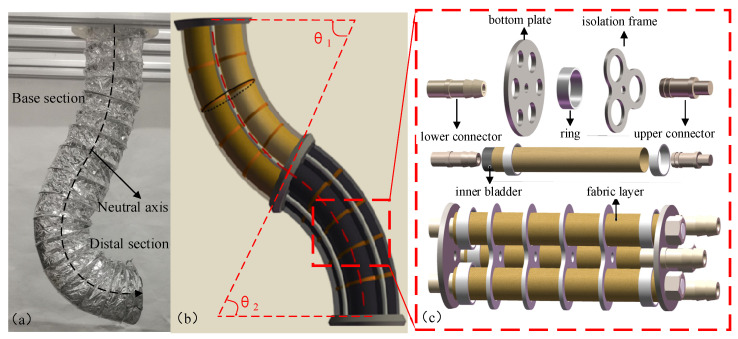
(**a**) Physical image of the soft manipulator. (**b**) Three-dimensional model of the soft manipulator. (**c**) Schematic diagram of the components of the soft manipulator.

In this paper, a fabric-enhanced soft manipulator driven by a water hydraulic system is presented as shown in Figure 1. The proposed soft manipulator is composed of two independent sections with six DOFs. For each section, three soft actuators are installed in parallel for flexible three-DOF movement in space, which not only ensures that the soft manipulator has sufficient degrees of freedom but also reduces the difficulty of its control. The soft actuators which are strengthened by the fabric layer, are fixed by the isolation frames and bottom plates.

**Figure 2:**
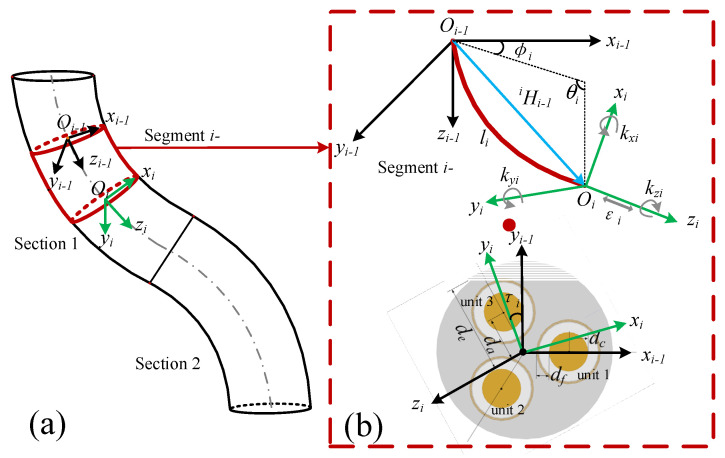
(**a**) Schematic diagram of the structure of two adjacent soft manipulators. (**b**) The shape parameters of the *i-th* segment of the soft manipulator and the coordinate transformation between two adjacent coordinate systems.

In order to make the model more accurate, we divide each section of the soft manipulator into *n* segments and one single segment is approximately regarded as a circular arc, as shown in Figure 2. Since the soft manipulator will not bear excessive shear stress in most cases, the discontinuity of the central axis curve caused by an excessive shear load is ignored when the soft manipulator is analyzed. Under these conditions, the attitude of segment *i-* can be represented by the length strain *ε_i_*, along the axis, the torsional strain, *k_zi_*, and the two normal bending strains, *k_xi_* and *k_yi_*. Then, the strain matrix of the soft segment *i-* is expressed as: [*ε_i_ kx_i_ ky_i_ kz_i_*]. We can use the various strain variables of each segment of the soft manipulator as the state variable calculated by the soft manipulator, and its state variable ***q*** is expressed as:

(1) $$\begin{array}{ll} q & ={\left[\begin{array}{cccc} q_{11} & q_{12} & \cdots & q_{n4} \end{array}\right]}^{T}\\ & ={\left[\begin{array}{cccc} q_{1} & q_{2} & \cdots & q_{4n} \end{array}\right]}^{T} \end{array}$$

where *q_ik_* represents the *k*-*th* state variable of the *i-th* segment, *i-*∈{1, 2, …, n}, *k-*∈{1, 2, 3, 4}, and *q_j_* represents the *j-th* state variable of all the segments, *j-*∈{1, 2, ..., 4n}.

As shown in Figure 2, where the arc length of the central axis of segment *i-* is *l_i_*, the bending angle is *θ_i_*, the deflection angle is *φ_i_*, and the torsion angle is *τ_i_*. The corresponding relationship between the strain variables and the shape parameters of the soft manipulator is as follows:

(2) $$\begin{array}{l} l_{i}=(1+\varepsilon_{i})\frac{l_{0}}{n}\\ \theta_{i}=l_{i}\sqrt{k_{xi}^{2}+k_{yi}^{2}}\\ \varphi_{i}=arc\tan2\left(-k_{xi},k_{yi}\right)\\ \tau_{i}=l_{i}k_{zi} \end{array}$$

For the proposed soft manipulator, we approximately treat each discrete soft manipulator as a circular arc of constant curvature. Then, we can extend the traditional D–H modeling method appropriately. The transformation between adjacent coordinate systems can be regarded as five rotations and one translation in a certain order.

Let *^b^*(**•**)*_a_* denote variables of the *a-th* frame expressed in the *b-th* frame, and let the zeroth frame be the robot base frame. According to the D–H method, the homogeneous transformation matrix between adjacent coordinate systems can be obtained as follows:

(3) Hi−1i=[cτ(cφ2(cθ−1)+1)+cφsφsτ(cθ−1)cφsφcτ(cθ−1)−sτ(cφ2(cθ−1)+1)cφsθ−licφ(cθ−1)θisτ(cθ−cφ2(cθ−1))+cφsφcτ(cθ−1)cτ(cθ−cφ2(cθ−1))−cφsφsτ(cθ−1)sφsθ−lisφ(cθ−1)θi−sφsτsθ−cφcτsθcφsτsθ−sφcτsθcθlisθθi0001]

(4) $$\begin{array}{ccc} c_{\varphi }=\cos\varphi_{i} & c_{\theta }=\cos\theta_{i} & c_{\tau }=\cos\tau_{i}\\ s_{\varphi }=\sin\varphi_{i} & s_{\theta }=\sin\theta_{i} & s_{\tau }=\sin\tau_{i} \end{array}$$

The homogeneous transformation matrix is introduced into the dynamics modeling of the soft manipulator to obtain the kinematic parameters of each segment. Firstly, the mass of each segment of the soft manipulator is approximately acts on the top center of each segment based on the lumped mass method. The angle of the soft manipulator in segment *i* in its local coordinate system is defined as:

(5) θi−1i=[−θi*sin(φi)θi*cos(φi)τi]

where *^i−1^**θ**_i_* represents the rotation angle of the soft manipulator in segment *i-* around the *x-*, *y-*, and *z-* axes, respectively, in its local coordinate system. Then, the angular velocity and angular acceleration of the soft manipulator in segment *i-* in its local coordinate system can be expressed as:

(6) ωi−1i=θ˙i−1iω˙i−1i=θ¨i−1i

Traditionally, the angular velocity and length of the connecting rod are generally used to calculate the magnitude of the linear velocity for the rigid manipulator. However, the geometry of the single segment is changes during the movement of the soft manipulator, so it is no longer suitable to calculate the linear velocity by using the angular velocity and length of the connecting rod. In this paper, the linear velocity of the segment is calculated directly by the derivation of the space position; the position of the mass point in the period of *i-* under the local coordinate system of its current segment can be expressed as: ***X****_i_* = [000]*^T^*. When performing coordinate transformation, the position vector is expanded as: ***γ*** *=* [***X****_i_^T^* 1]*^T^*. Through homogeneous transformation, the position of segment *i*-1 can be obtained as:

(7) Xi−1i=gHi−1iγ

(8) $$g=\left[\begin{array}{cccc} 1 & 0 & 0 & 0\\ 0 & 1 & 0 & 0\\ 0 & 0 & 1 & 0 \end{array}\right]$$

It is concluded that the partial derivative of the mass point position vector of the soft manipulator in segment *i-* is defined as:

(9) ∂Xi−1i∂qik=∂(gHi−1iγ)∂qik=g∂(Hi−1i)∂qikγ+gHi−1i∂(γ)∂qik⏟=0+∂(g)∂qik⏟=0·Hi−1iγ

Then, its velocity in the current local coordinate system can be expressed as:

(10) vi−1i=dXi−1idt=ddt(gHi−1iγ)=(gH˙i−1iγ+gHi−1idγdt⏟=0+dgdt⏟=0·Hi−1iγ)

(11) H˙i−1i=∑ξ=1i∑χ=14∂H˙i−1i∂qξχq˙ξχ

The derivative of the velocity of the soft manipulator and its acceleration can be expressed as:

(12) v˙i−1i=d(gH˙i−1iγ)dt=gH¨i−1iγ=g·∑ξ=1i∑χ=14(∂Hi−1i∂qξχq¨ξχ+∑α=1i∑β=14∂2Hi−1i∂qαβ∂qξχq˙αβq˙ξχ)·γ

### 2.2. Dynamics

**Figure 3:**
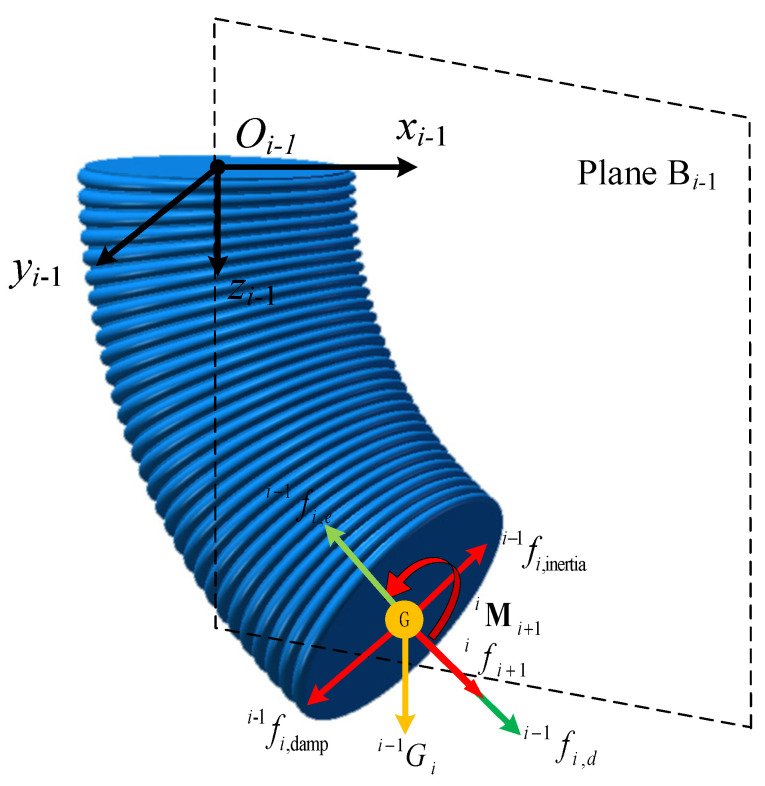
Force diagram of the soft manipulator in segment *i*.

According to the Newton-Euler iterative method, the equilibrium of each segment of the soft manipulator is analyzed. In contrast to the modeling of rigid robots, external force will cause the shape of the soft manipulator to change, while the tiny deformation of the connecting rod is ignored. However, different stress conditions can cause different degrees of deformation of the soft manipulator, and part of the energy of the external force and driving force is used to offset the elastic deformation of the soft manipulator. Therefore, the force of the soft manipulator in segment *i-* is shown in Figure 3. According to the force diagram, the force balance and torque balance equations of the soft manipulator in segment *i-* are expressed as follows:

(13) Fi=fi−1i,e+fi−1i,d+Gi−1i+fi−1i,damp+fi−1i,inertia+Ri−1ifii+1Mi=Mi−1i,e+Mi−1i,d+Mi−1i,G+Mi−1i,damp+Mi−1i,inertia+Xi−1i×Ri−1ifii+1+Ri−1iMii+1

where, ***F****_i_* is the resultant force of the soft manipulator in segment *i,* and ***M****_i_* is the combined torque of the soft manipulator in segment *i-.* Additionally, the resultant force and moment of the soft manipulator in segment *i-* is zero. The expressions of the forces and torques will be described in detail below.

#### 2.2.1. Inertial Force and Moment

The inertial force, *^i−1^**f**_i,inertia_*, and moment, *^i−1^**M**_i,inertia_*, of segment *i-* in the local coordinate system, *o_i-1_*, are obtained as follows:

(14) fi−1i,inertia=miv˙i−1iMi−1i,inertia=Iiω˙i−1i+ωi−1i×Iiωi−1i

where, *m_i_* is the mass of segment *i-*, and ***I****_i_* is the inertia tensor of segment *i-.* According to the geometrical shape of the soft manipulator, the inertia tensor, ***I****_i_*, can be expressed as:

(15) $$I_{i}=\left[\begin{array}{ccc} I_{i,x} & & \\ & I_{i,y} & \\ & & I_{i,z} \end{array}\right]$$

With the moments of inertia defined as:

(16) $$\begin{array}{l} I_{i,x}=I_{i,y}=\frac{1}{4}m_{i}r_{i}^{2}\\ I_{i,z}=\frac{1}{2}m_{i}r_{i}^{2} \end{array}$$

where, *r_i_* represents the radius of the soft manipulator.

#### 2.2.2. Elastic Force and Moment

For soft materials that can deform in a compliant manner, the magnitude of the elastic force and moment is only related to the degree of deformation in the previous state. Therefore, the elastic force, *^i−1^**f**_i,e_*, and the elastic moment, *^i−1^**M**_i,e_*, of the soft manipulator in segment *i-* are only related to its own expansion. According to the material elastic deformation formula, the elastic force of the soft manipulator in its current state can be expressed as:

(17) fi−1i,e=[00EAs∑j=13(lij−li0)]T

where *E* is the elastic modulus of the soft manipulator, *A_s_* is the cross-sectional area of the annular entity of the soft unit, *l_ij_* is the arc length of the central axis of the *j-th* soft unit of segment *i-,* and *l_i0_* is the arc length of the central axis of segment *i-.*

According to the relationship between stress and strain in the constitutive model of the viscoelastic rod, the relationship between the bending strain generated by the rotation of the soft element around the *x-* and *y-* axes and the torsional strain generated by the rotation around the *z-* axis and torque is as follows:

(18) Mi−1i,e=Ki[kxikyikzi]T

(19) $$K_{i}=diag\left(\left[\begin{array}{ccc} EI_{xxi} & EI_{yyi} & GI_{zzi} \end{array}\right]\right)$$

where, *I_xxi_* = *I_yyi_* = *πr_i_*^4^/4 and *I_zzi_* = *πr_i_*^4^/2 denote the area moments of the inertial and polar moments of inertia for the cross segment, respectively. ***K****_i_* is the stiffness coefficient of the soft manipulator in segment *I*, *E* is the elastic modulus of the soft manipulator, and *GI_zzi_* is the torsional stiffness of the soft manipulator.

#### 2.2.3. Driving Force and Torque

In this paper, a hydraulic system is used to actuate the soft manipulator, so the driving force and torque of the soft unit are only determined by the driving pressure inside the soft cavity. In the local coordinate system where each discrete segment of the soft manipulator is located, the direction of driving pressure always acts along the axis of the soft unit. According to the driving pressure and the effective action area inside the soft unit, the driving force vector of the soft manipulator in segment *i* in its local coordinate system can be expressed as:

(20) fi−1i,d=[00Ad∑j=13Pi,j]T

where, *P_i,j_* represents the driving pressure of the *j-th* soft unit in segment *i-* (the soft manipulator consists of three soft units in parallel) and *A_d_* represents the effective area inside the soft unit. The driving moment of the soft manipulator in segment *i-* is the combined moment of three soft units. Its vector expression is as follows:

(21) Mi−1i,d=∑j=13(rd,j×fi,j,d)

where ***f****_i,j,d_* represents the driving force of the *j-th* soft unit of segment *i-* and ***r****_d,j_* represents the vector diameter of the *j-th* soft unit.

#### 2.2.4. Interaction between Soft Units

We analyze the interaction forces between adjacent soft manipulators. Firstly, the soft manipulator, without segment *n*, is analyzed. As a soft manipulator made of elastic material, the force on both ends of each segment of the soft manipulator is not transmitted directly as in a rigid manipulator, but the force acting on the end of this segment of the soft manipulator is transmitted through the elastic force of the elastomer. For the soft manipulator, the driving force is its internal force. Therefore, the force, *^i^**f**_i+1_*, and the acting moment, *^i^**M**_i+1_*, transmitted by the back and forward segment can be expressed as:

(22) fii+1=fii+1,e−fii+1,dMii+1=Mii+1,e−Mii+1,d

where, i∈{1,…,n−1}.

When the soft manipulator of segment *n* is analyzed, it is assumed that the end of the soft manipulator is subjected to the external load force and external load moment exerted by the external environment. These are represented by *^n^**f**_load_* and *^n^**M**_load_* in the current local coordinate system. Therefore, the force *^n^**f**_load_* and the acting moment *^n^**M**_load_* of the soft manipulator in segment *n* can be expressed as:

(23) fnn+1=fnloadMnn+1=Mnload

#### 2.2.5. Damping Force and Moment

The soft manipulator will be affected by damping due to its own flexible materials, elastic fabrics, and the environment during actual movement. The damping force received by the soft manipulator is related to its motion speed. The damping force received by the soft manipulator in segment *i* can be expressed as follows in its local coordinate system:

(24) [fi−1i,dampMi−1i,damp]=[dvvi−1idωωi−1i]

where, *d_v_* and *d_ω_* represent the motion damping coefficient and rotational damping coefficient of the soft manipulator, respectively.

#### 2.2.6. Gravity

The gravity of the soft manipulator in segment *i-* acts on the concentrated mass point at the top center of the manipulator. Additionally, the direction is always vertically downward, as shown in Figure 3. The torque generated by gravity can be expressed as:

(25) Mi−1i,G=Xi−1i×Gi−1i

where, *^i−1^**X**_i_* is the position coordinate of the mass point of the soft manipulator in segment *i-*.

Using the formulas above, the states of all the discrete segments of the soft manipulator can be obtained. Because the shear effect generated by the soft manipulator in the process of motion is ignored, only the force along the z axis is considered in the calculation of the force. Therefore, the dynamic model of the soft manipulator can be expressed as:

(26) $$M\left(q\right)\ddot{q}+C\left(q,\dot{q}\right)\dot{q}+N\left(q\right)=\tau \left(p\right)$$

where, ***M***(***q***) is the inertia matrix, which can be expressed as:

(27) M(q)=[−Z^mg∂Hi−1i∂qγ−Xi−1i×(mg∂Hi−1i∂qγ)−IiRi−1i∂ωi−1i∂q]

where $\hat{Z}=\left[\begin{array}{ccc} 0 & 0 & 1 \end{array}\right]$.

$C(q,\dot{q})$ represents the Coriolis force and damping force matrix, which can be expressed as:

(28) C(q,q˙)=[−Z^(mg·∑α=1i∑β=14∂2Hi−1i∂q∂qαβq˙αβ+dv∂Hi−1i∂q)γ−(U×IiU+(dω+Ii)·∑ζ=1iRi−1ζ−1∂θi−1i∂q)]

(29) U=∑ζ=1iRi−1ζ−1∂θi−1i∂q

***N***(***q***) is a composite matrix, including mass force, elastic force, and the interaction force between adjacent soft manipulators. It can be expressed as:

(30) N(q)=[Z^(Ri−1i−1)−1Gi−1iXi−1i×((Ri−1i−1)−1Gi−1i)]+[Z^Ri−1ifii+1Xi−1i×Ri−1ifii+1+Ri−1iMii+1]−Kqr

***τ***(***p***) includes the driving force and external load influence, which can be expressed as:

(31) τ(p)=[Z^fi−1i,dMi−1i,d]+[(Ri−1i−1)−1Fext(Ri−1i−1)−1Mext]

### 2.3. Dynamics of the Water Hydraulic System

**Figure 4:**
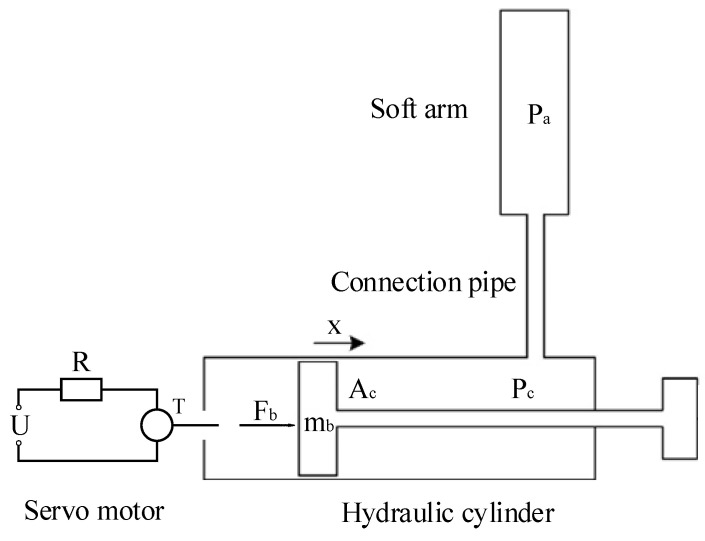
Schematic diagram of the hydraulic system for driving the soft manipulator. At the left is the schematic representation of the servo motor, and at the right is the representation of the piston and hydraulic cylinder. Above is a schematic representation of the soft manipulator and connecting pipes.

In order to actuate each soft unit of the soft manipulator independently, a water hydraulic system was developed. As shown in Figure 4, the screw is actuated to rotate by controlling the rotation of the servo motor. The end of the screw was firmly connected with the piston of the hydraulic cylinder. The piston of the hydraulic cylinder can move linearly. The fluid in the hydraulic cylinder flows into the cavity of the soft unit under the action of pressure. In addition, each actuator in the water hydraulic system only actuates one soft unit.

In the closed space composed of the hydraulic cylinder and soft unit, the number of fluid molecules remains unchanged and the volume changes, so it can achieve the purpose of changing the pressure. According to the dynamic model of the soft manipulator, the attitude change of the soft manipulator can be predicted under a given fluid-driven pressure change. In the actual driving process of the hydraulic cylinder driven by the servo motor-ball screw, there is a certain delay effect in the change of the motor rotation on the actual pressure. Therefore, understanding the dynamic characteristics of the hydraulic system is of guiding significance to the decision-making of the soft robot’s drive signal input. The equation of the hydraulic cylinder is as follows:

(32) $$F_{b}=m_{b}\ddot{x}+f+P_{c}A_{c}+d_{v}\dot{x}$$

where *F_b_* is the force exerted by the screw on the piston, *P_c_* is the pressure inside the hydraulic cylinder, *d_v_* represents the damping coefficient, and *A_c_* and *m_b_* are the cross-sectional area and mass of the piston, respectively. Lastly, *x* is the piston displacement. The relationship between the motor torque and the force acting on the piston are as follows:

(33) $$\begin{array}{l} \eta T\omega =F_{b}\cdot \dot{x}\\ \dot{x}=\omega \cdot s \end{array}$$

where *T* is the motor torque, *η* represents the power loss coefficient and *s* represents the lead of the lead screw.

Because there is a certain pressure transfer delay between the inside of the cylinder and the soft manipulator (refer to the paper by Rus et al. [34]), the fluid flowing into the soft cavity can be expressed as:

(34) $$\frac{\pi d_{p}{}^{4}(P_{c}-P_{a})}{128\mu l_{p}}=\left(\frac{A_{d}{}^{2}l_{0}}{EA_{s}}+\frac{V_{1}}{K}\right){\dot{P}}_{a}$$

where, *P_a_* represents the internal pressure of the soft manipulator, *d_p_* and *l_p_* are the diameter and length of the connecting tube, and *μ* is the absolute viscosity of the fluid. *K* represents the bulk modulus of water, *l*_0_ is the initial length of the soft manipulator, and *V*_1_ represents the initial volume of the soft manipulator. The fluid flowing into the hydraulic cylinder can be expressed as:

(35) $$A_{c}\dot{x}+\frac{\pi d_{p}{}^{4}(P_{a}-P_{c})}{128\mu l_{p}}=\frac{V_{2}}{K}{\dot{P}}_{c}$$

where *V*_2_ is the initial volume of the hydraulic cylinder. The corresponding relationship between the motor torque and the internal driving pressure of the soft unit can be obtained by the simultaneous use of Equations (29)–(32), as follows:

(36) $$\left[\begin{array}{l} \ddot{x}\\ {\dot{P}}_{a}\\ {\dot{P}}_{c} \end{array}\right]=\left[\begin{array}{ccc} -\frac{d_{v}}{m_{b}} & 0 & -\frac{A_{c}}{m_{b}}\\ 0 & -\frac{\pi d_{p}{}^{4}}{128\mu l_{p}\left(\frac{A_{d}{}^{2}l_{0}}{EA_{s}}+\frac{V_{1}}{K}\right)} & \frac{\pi d_{p}{}^{4}}{128\mu l_{p}\left(\frac{A_{d}{}^{2}l_{0}}{EA_{s}}+\frac{V_{1}}{K}\right)}\\ \frac{A_{c}K}{V_{2}} & \frac{\pi d_{p}{}^{4}K}{128\mu l_{p}V_{2}} & -\frac{\pi d_{p}{}^{4}K}{128\mu l_{p}V_{2}} \end{array}\right]\cdot \left[\begin{array}{c} \dot{x}\\ P_{a}\\ P_{c} \end{array}\right]+\left[\begin{array}{c} \frac{\eta }{sm_{b}}\\ 0\\ 0 \end{array}\right]T-\left[\begin{array}{c} fm_{b}\\ 0\\ 0 \end{array}\right]$$

The relevant calculation parameters of the soft manipulator and the hydraulic system model are shown in Table 1.

## 3. Simulation Results

### 3.1. Workspace

**Figure 5:**
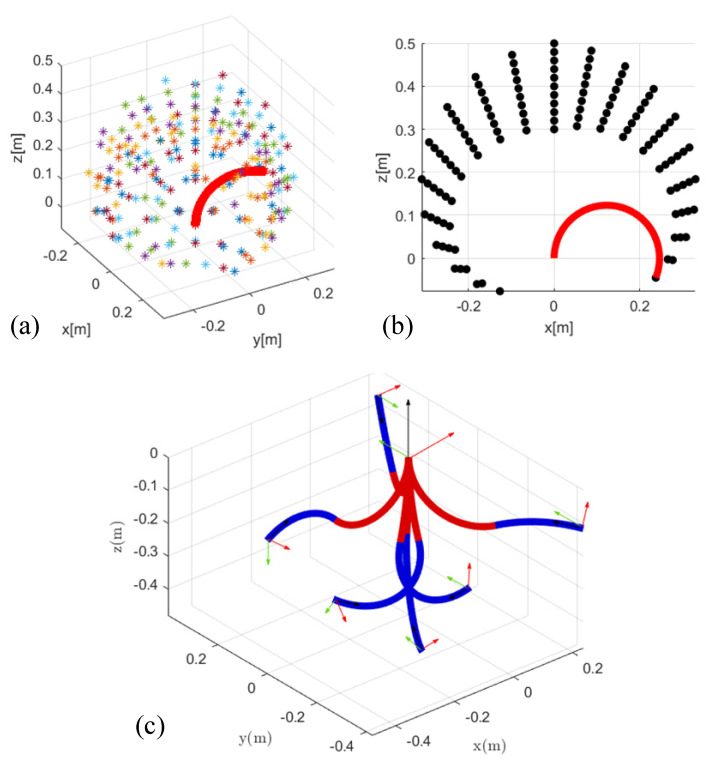
(**a**) The 3D working space of the single-section soft manipulator. (**b**) The bending state of the single-section soft manipulator in the XOZ plane. (**c**) The limit posture of the soft manipulator in space when the pressure on each soft unit of the two-section soft manipulator reaches the limit value (1 mpa).

In order to reduce the amount of calculation and increase the simulation speed, we simply treat each section of the soft manipulator as a single arc. In other words, when simulating the soft manipulator, we regard each section of the soft manipulator as only one segment. In this part, we will simulate and analyze the proposed dynamic model of the soft manipulator. The analysis of the workspace of the single-section soft manipulator has guiding significance for analyzing the execution ability of the soft manipulator in different environments. We call the collection of end points of the soft manipulator’s motion as the workspace. For a single-section soft manipulator, by changing the driving pressure inside the three soft cavities (within the safety pressure range of the soft manipulator 0-1 MPa), the corresponding workspace is obtained as a spherical shell, as shown in Figure 5a. Its workspace is approximately 0.5 m × 0.5 m × 0.5 m. We analyze its workspace in the XOZ plane as shown in Figure 5b. Because the three-cavity separation uniformly distributed non-axisymmetric structures adopted in this paper, there is a certain difference in the two-way bending performance of the soft manipulator. When the torque of the first, second and third actuators of the water hydraulic system is 0 N·m, 2.5 N·m, and 2.5 N·m (at this time, the second and third soft units are actuated at the same time at the maximum pressure and the first soft unit is treated with atmospheric constant pressure; *P*_2_ = *P*_3_ = 1 MPa, *P*_1_ = 0 MPa), the soft manipulator reaches the x-axis positive bending limit, its bending angle can reach 165.7°, the axis arc length is 0.401 m, and the radius of the curvature is 0.139 m. When the torque of the first, second and third actuators of the water hydraulic system is 2.5 N·m, 0 N·m, and 0 N·m (at this time, the first soft unit is actuated at the maximum pressure and the other two soft units are subjected to constant pressure treatment; *P*_2_ = *P*_3_ = 0 MPa, *P*_1_ = 1 MPa), the soft manipulator reaches the x-axis negative bending limit, and its bending angle is 168 °, the arc length of the axis is 0.473 m, and the radius of the curvature is 0.136 m. It can be seen that the bending angle of the soft manipulator is larger when it is actuated unilaterally. After the single-section soft manipulator is simulated and analyzed, the two-section soft manipulator model is simulated and analyzed. Figure 5c shows the limit posture of the soft manipulator in the three-dimensional space when the ultimate pressure (1 MPa) that it can bear is applied to each soft unit of the soft manipulator.

### 3.2. Results

#### 3.2.1. Unidirectional Bending with Step Input Torque

**Figure 6:**
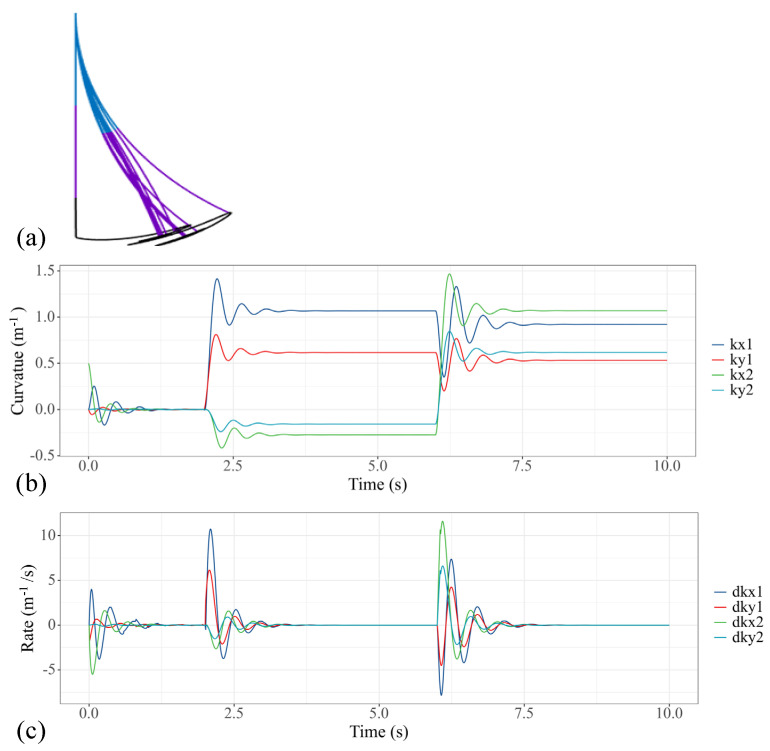
Simulation results of the soft manipulator when the first, fifth and sixth actuators are actuated by step torque. (**a**) Soft manipulator trajectory versus time. (**b**) Curves of the bending curvature versus time. (**c**) Curves of speed versus time.

The performance of the modeling method presented in this study is rooted in the fact that not only the static solution but also the dynamic trajectory can be obtained. In this case, we respectively provide 1.5 N·m step torque to the first, fifth and sixth actuators of the water hydraulic system, so that the two sections of the soft manipulators can bend in the same direction and the motion of the theoretical model is in a plane. Figure 6 shows the converging process of the soft manipulator model to a stable state. Figure 6a shows the trajectory change of the soft manipulator. Figure 6b shows how the curvature of the soft manipulator changes to the given step input, while Figure 6c shows the changing trend of the speeds. The steady state is obtained within 1 s after the soft manipulator is actuated.

#### 3.2.2. Bi-Directional Bending with Step Input Torque

**Figure 7:**
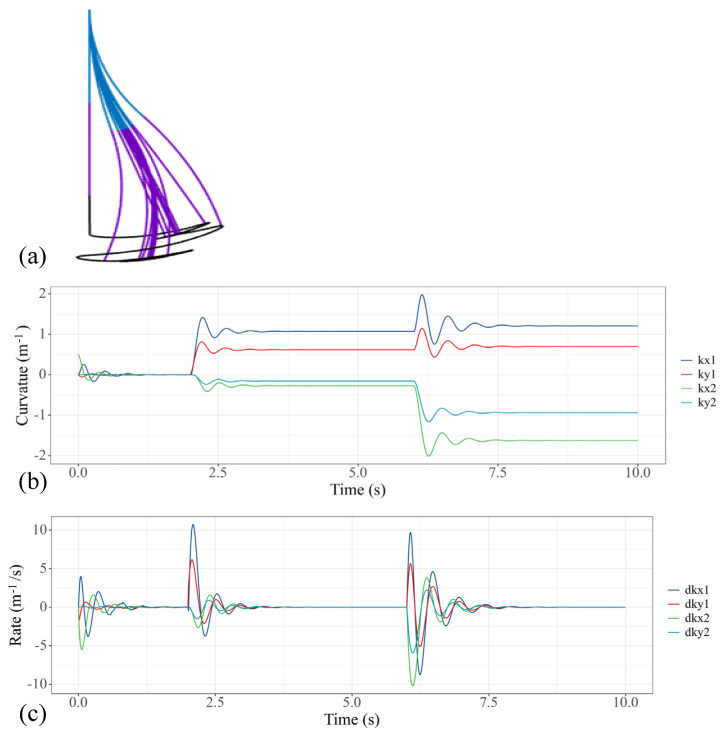
Simulation results of the soft manipulator when the first and fourth actuators are actuated by step torque. (**a**) Soft manipulator trajectory versus time. (**b**) Curves of the bending curvature versus time. (**c**) Curves of speed versus time.

In this case, we respectively provide 1.5N·m step torque to the first, fifth and sixth actuators of the water hydraulic system, so that the two sections of soft manipulators can be bent in reverse. The performance of the model is shown in Figure 7. Figure 7a shows the trajectory change of the soft manipulator. Figure 7b shows how the curvature of the soft manipulator changes to the given step input, while Figure 7c shows the changing trend of the speeds. The steady state is obtained within 1.5 s after the soft manipulator is actuated.

#### 3.2.3. Unidirectional Bending with Ramp Input Torque

**Figure 8:**
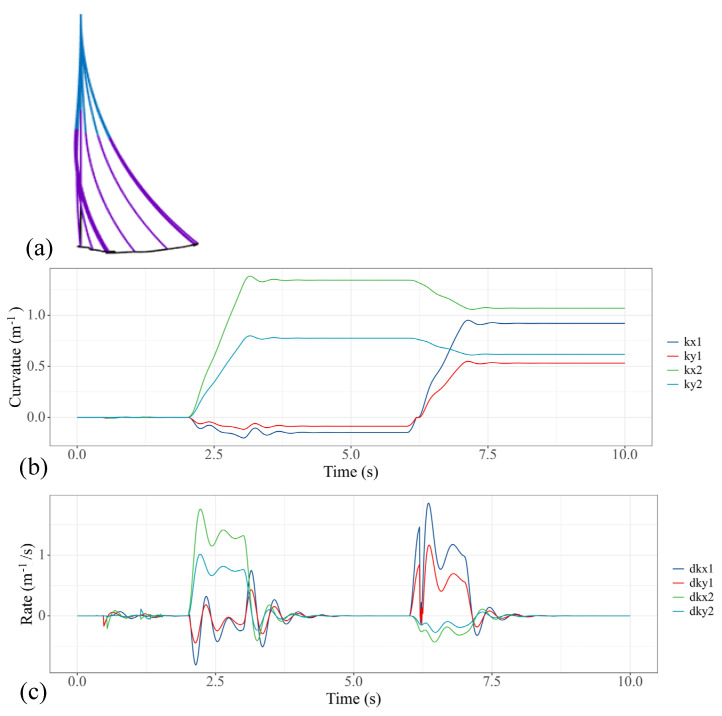
Simulation results of the soft manipulator when the first, fifth and sixth actuators are actuated by ramp torque. (**a**) Soft manipulator trajectory versus time. (**b**) Curves of the bending curvature versus time. (**c**) Curves of speed versus time.

In practical applications, we often gradually increase the pulling torque applied on the soft manipulator. In this case, we actuated the first, fifth and sixth actuators with a time-variant torque, which can be expressed as a function of time: *T_input,_*_1_ = 1.5(*t*-6) (6 s < *t* < 7 s), *T_input,_*_5_ = 1.5(*t*-2) (2 s < *t* < 3 s) and *T_input,_*_6_ = 1.5(*t*-2) (2 s < *t* < 3 s). It can be noted that, with the ramp input of the torque, the oscillation of the curvatures as well as the speeds is significantly reduced, as shown in Figure 8. Because the motor torque is gradually increasing, the pressure applied to the soft manipulator will not produce a sudden change; therefore, the dynamic model of the soft manipulator can maintain a relatively stable speed without too much oscillation.

## 4. Experimental Results

### 4.1. Test Platform

**Figure 9:**
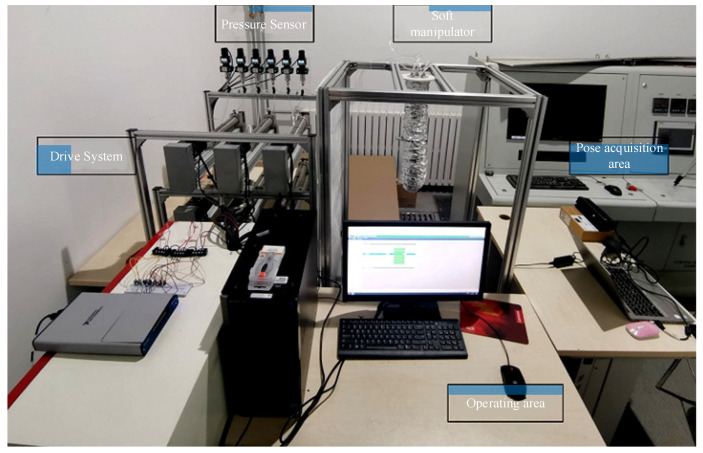
Soft manipulator test platform.

In order to independently control each soft unit of the soft manipulator and improve the performance of the soft manipulator, a test platform was built, as shown in Figure 9. The test platform is mainly composed of a computer, the water hydraulic system, and an information acquisition system. The proposed water hydraulic system is mainly composed of six servo motors and six hydraulic cylinders. We use a Yaskawa controller with the type of MP2100 to control the servo motors, and we develop a motor torque control algorithm based on the software MPE720. The information acquisition system can obtain the soft manipulator posture, water pressure, motor torque and speed, which can be used to analyze the characteristics of the soft manipulator. We obtain the pressure data through the HH316 pressure sensors and the data acquisition board (DAQ) is chosen as USB-6341. The posture of the soft manipulator was recorded using D4 cameras.

Simulations were carried out in the joint space for the input torque profiles associated with the experiments. The joint-space trajectories data are recorded 100 times per second from the simulations. Then, the resulting joint space trajectories are transformed to task-space trajectories of each section tip (*ψ*_1_ and *ψ*_2_) by Equation (2) for all *i* = 1, 2. Due to the redundancy of the soft manipulator, it is important to establish the uniqueness of compared arm poses. For a given end effector position, the soft manipulator model is verified against the unique continuous arm posture without redundant conflicts. It can be thought of as a two-point shape estimation of the entire soft manipulator, and conforms to the shape accuracy measuring requirements. Hence, the two uniformly distributed section tip positions of the prototype arm are compared against the corresponding section tip positions of the dynamic model. To quantify the agreement between the experimental and simulation data, the instantaneous error is defined as:

(37) $$ERR_{i}=∥\psi_{i}{}^{e}(t)-\psi_{i}(t)∥=\sqrt{{\left(x_{i}{}^{e}(t)-x_{i}(t)\right)}^{2}+{\left(y_{i}{}^{e}(t)-y_{i}(t)\right)}^{2}+{\left(z_{i}{}^{e}(t)-z_{i}(t)\right)}^{2}}$$

where *i-* is the section number (*I* = 1, 2), and the instantaneous error is computed at each section tip and plotted along with task-space coordinates. Finally, in order to quantify the overall model accuracy, the maximum of the mean cumulative error of the arm and the maximum sectional error are computed for each experiment. The maximum cumulative error is defined as max $\left(\sum_{i=1}^{2}ERR_{i}\right)$ and the maximum sectional error is given by max ([max (*ERR*_1_), max (*ERR*_2_)]).

### 4.2. Steady Performance

**Figure 10:**
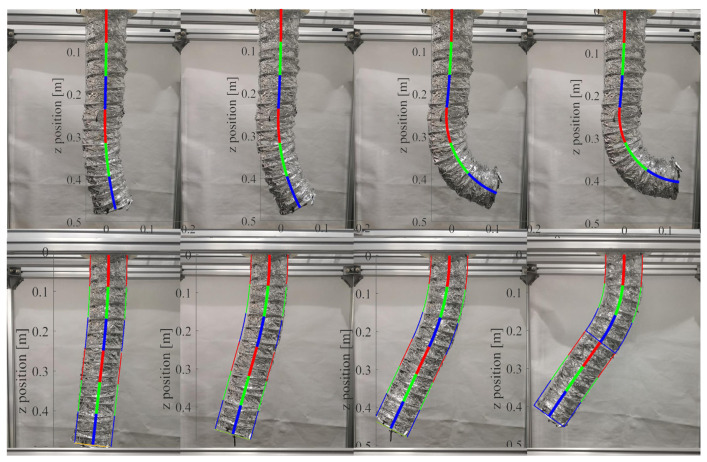
The steady state posture of the soft manipulator under different driving pressures.

**Figure 11:**
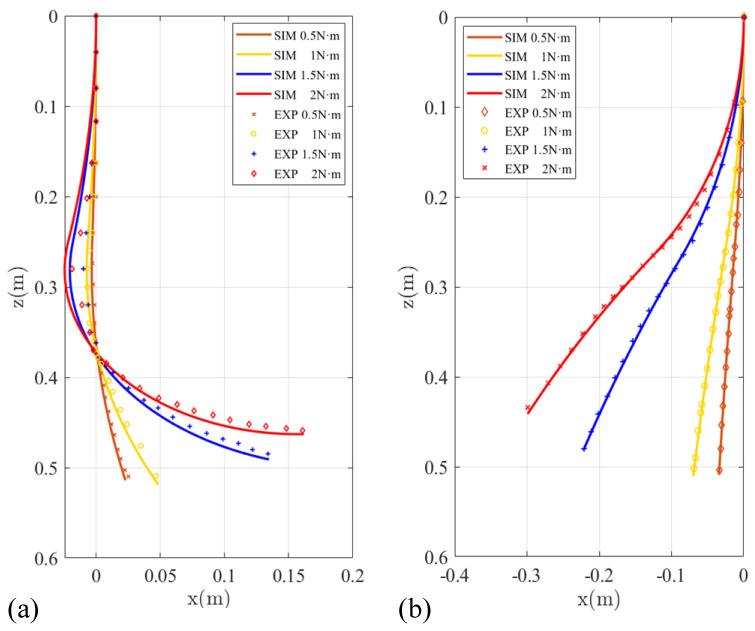
(**a**) The comparison of experimental results and simulation results when the second section of the soft manipulator is actuated by different torque to reach a steady state. (**b**) The comparison of experimental results and simulation results when the first section of the soft manipulator is actuated by different torque to reach a steady state.

Due to the flexible deformability, the soft manipulator can adapt to the tortuous path by shaping their body according to specific environmental requirements. Here, we first validate the steady performance of the proposed model. The theoretical spatial poses were calculated numerically, and the simulation results of curvatures were compared with those measured photographically. In order to facilitate the measurement and verification, we mainly study the plane stability performance of the soft manipulator. We separately apply pressures of 0.5 N·m, 1 N·m, 1.5 N·m, and 2 N·m to the first or fourth actuators of the water hydraulic system to make the first section or the second section of the soft manipulator move independently. During the experiment, the stable posture of the soft manipulator at different torque is shown in Figure 10. Figure 11a shows the comparison between the model shape of the soft manipulator and the experimental shape when the second section of the soft manipulator is actuated to a stable state with different torque. In Figure 11a, it can be seen that when a torque of 0.5 N·m or 1 N·m is input to the dynamic model, the model shape of the soft manipulator fits well with the experimental shape. When the step torque of 1.5 N·m or 2 N·m is input to the dynamic model, the experimental results are different from the theoretical results. The possible reason for this is that the instantaneous speed of the soft manipulator will be high when large torque is input, which will affect the experimental results.

Figure 11b shows the comparison between the experimental results and the simulation results when the first section of the soft manipulator is actuated to a stable state with different torque. It can be seen from the figure that the experimental results are not much different from the simulation results. This is because when the first section of the soft manipulator is actuated to move, the second section of the soft manipulator has a greater resistance, which can reduce the impact and make the simulation and experiment fit well. A comprehensive comparison of Figure 11 shows that the proposed soft manipulator model has better steady performance.

### 4.3. Dynamic Performance

Because the structure of the soft manipulator is symmetrical, in order to conveniently measure and verify the dynamic performance of the soft manipulator, we only actuate the soft manipulator to move in the XOZ plane.

#### 4.3.1. Step Input Torque

**Figure 12:**
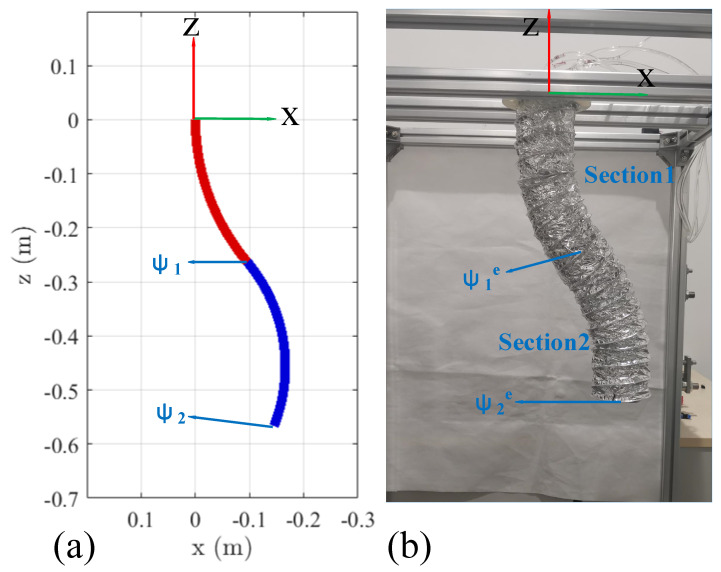
Final pose comparison of the dynamic model and prototype continuum arm for in plane bending of all sections. (**a**) The final posture of the soft manipulator simulation model. (**b**) The final posture of the prototype continuous arm.

**Figure 13:**
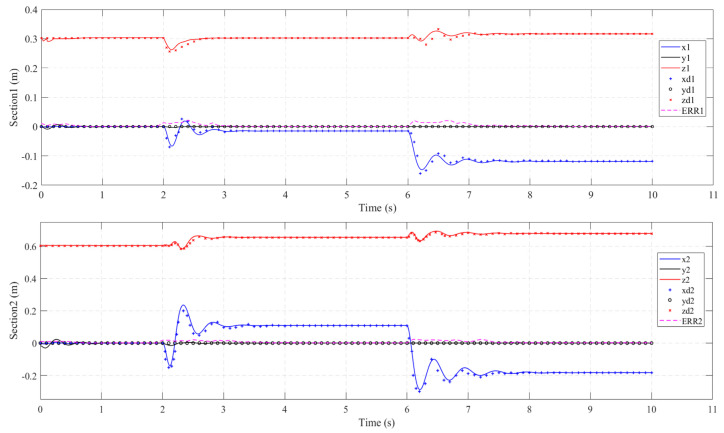
Sequential planar bending of all the sections. Comparison of the dynamic section tip coordinate evolution (solid line) vs. the corresponding coordinate values of the prototype arm (X, Y, and Z experimental data are denoted by o, +, and × marks, respectively). Position errors, *ERR_i_* = ||*ψ_i_^e^*-*ψ_i_*|| ∀*i* ϵ {1,2}, are denoted by a dashed line in each subplot.

In this experiment, we analyze the dynamic performance of the soft manipulator model when step torque is input. By actuating the soft manipulator separately, both the passive and active responses of the arm can be clearly compared. The fourth and first actuators of the water hydraulic system are provided with 1.5 N·m step torque input at *t* = 2 s and *t* = 6 s, respectively, and maintained throughout the experiment. As described in Section 3, these torque commands cause the soft manipulator to bend in the XOZ plane. The dynamic model is also given the same torque command sequence and the tip coordinate values are compared in Figure 12. Each subplot denotes the tip coordinates of the base (Section 1, *ψ*_e1_) and distal (Section 2, *ψ*_e2_) of the soft manipulator, respectively, along with the relevant section tip position errors. The corresponding visual comparison of the prototype and simulation model poses at *t* = 10s is shown in Figure 13.

From the comparison of the output data and the error, it can be seen that the proposed dynamic model of the soft manipulator is in good agreement with the experimental data. The maximum sectional error was 0.0305 m and the maximum cumulative error was 0.0565 m. Through careful inspection, we can find that significant deviations in the position error (*ERR*_1_ and *ERR*_2_) of the soft manipulators at both ends were observed during the step input, and that these deviations quickly decayed to a steady-state value.

#### 4.3.2. Ramp Input Torque

**Figure 14:**
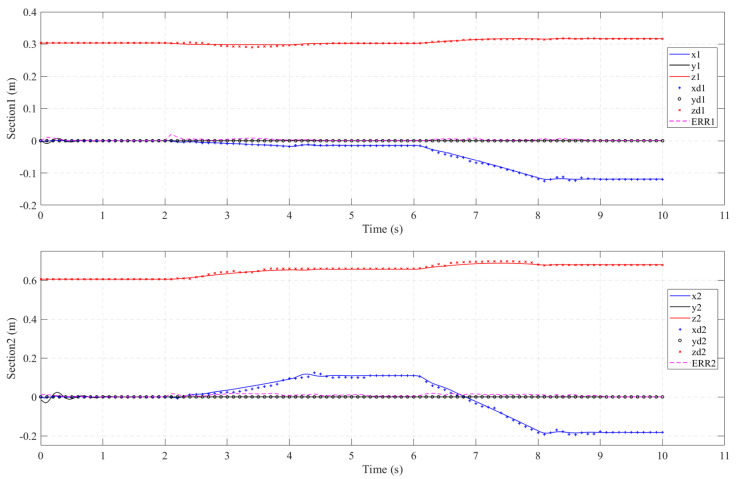
Sequential planar bending of all the sections. Comparison of the dynamic section tip coordinate evolution (solid line) vs. the corresponding coordinate values of the prototype arm (X, Y, and Z experimental data are denoted by o, +, and × marks, respectively). Position errors, *ERR_i_* = ||*ψ_i_^e^*-*ψ_i_*|| ∀*i* ϵ {1,2}, are denoted by a dashed line in each subplot.

In practical situations, we often gradually increase the input torque in the dynamic model. Therefore, in this experiment, the first and fourth actuators of the water hydraulic system are inputted with ramp torque and the torque can be expressed as: *T_input,_*_1_ = 0.75(*t*-6) (6 < *t* < 8); *T_input,_*_4_ = 0.75(*t*-2) (2 < *t* < 4). Additionally, we input the same driving conditions in the simulation model. The tip coordinate values are compared in Figure 14. Each subplot denotes the tip coordinates of the base (Section 1, *ψ*_e1_) and distal (Section 2, *ψ*_e2_) of the soft manipulator, respectively, along with the relevant section tip position errors. From the comparison of Figure 13 and Figure 14, it can be seen that when the torque is gradually input to the soft manipulator, the end coordinates of the soft manipulators change more smoothly and that there is no oscillation phenomenon.

As shown in Figure 14, it also can be seen that the proposed dynamic model of the soft manipulator is in good agreement with the experimental data. The maximum sectional error was 0.0245m and the maximum cumulative error was 0.042m. Through careful inspection, we can find that when the ramp pressure is input to the soft manipulator, its end position error (*ERR*_1_ and *ERR*_2_) changes smoothly and the peak value of the error fluctuation is small.

## 5. Conclusions

In this paper, we take the analysis of the motion posture of the fluid-driven soft manipulator as a starting point. Then, we establish a dynamic model of the soft manipulator based on the Newton-Euler iterative method, and we build a hydraulically driven experimental system. Finally, the accuracy of the model is verified through experiments.

The contributions are summarized in the following points. Firstly, the dynamic model of the soft manipulator is established based on an improved Newton-Euler iterative method, which comprehensively considers the influence of inertial force, elastic force, damping force and combined bending and torsion moments. Additionally we also have established a dynamic model of a water hydraulic system, which comprehensively considers the influence of cylinder inertia, friction, and water hydraulic response. Secondly, we built a complete soft manipulator test system. According to the actuation requirements of fluid-driven soft manipulators, a water hydraulic system was built. At the same time, in order to facilitate the posture analysis of the soft manipulator, a complete data acquisition system was built, which can realize all-round collection of the displacement, speed, motor torque, soft manipulator pressure and posture information of the hydraulic cylinder. Thirdly, we completed the steady-state bending posture test for the soft manipulator based on the experimental platform. By analyzing the simulation results and experimental results, it can be known that under different pressures, the simulation results of the soft manipulator’s steady-state pose and the experimental results are slightly different, and that the steady performance of the soft manipulator’s dynamic model is good. In addition, we tested the dynamic characteristics of the soft manipulator driven by step torque and ramp torque, respectively. By collecting and comparing the coordinates of the end of the soft manipulator, we found that the maximum sectional error of the soft manipulator is 0.0305 m when driven by step torque and that the maximum sectional error of the soft manipulator is 0.0245 m when driven by ramp torque. It can be seen that the dynamic error of the soft manipulator is small and that the model has better dynamic characteristics. The above experiments and simulation analysis verify the accuracy and validity of the dynamic model proposed in this paper.

Because the soft manipulator has infinite degrees of freedom, the current dynamic models still have unmodeled states. Therefore, we can improve the overall performance of the soft manipulator by stimulating the hidden dynamics [35]. For future research on the dynamic model of the soft manipulator, an important aspect is to develop a theory that it is more suitable for an imperfect system with higher computational efficiency and develop a novel control strategy for an imperfect model. In this paper, we developed a water hydraulic system, which actuates the soft manipulator by a hydraulic cylinder. In future work, we will develop a microfluidic actuation system that actuates the soft manipulator through the cooperation of hydraulic pumps and valves [36]. The volume of this microfluidic system is smaller and it has better dynamic characteristics.

## Figures and Tables

**Table 1:** Relevant Parameters.

Symbol	Value
*l_0_*	0.25 m
*E*	1.2 MPa
*K*	2.18 × 10^9^ Pa
*A_d_*	3.14 × 10^−4^ m^2^
*A_c_*	0.0021 m^2^
*A_s_*	3.93 × 10^−4^ m^2^
*l_p_*	1.61 m
*η*	0.9
*m_b_*	1.2 kg
*s*	4 mm
*μ*	1.01 × 10^−3^ Pa·s
*V_1_*	7.85 × 10^−5^ m^3^
*V_2_*	7.35 × 10^−4^ m^3^
*d_p_*	0.004 m

## Data Availability

Not applicable.
